# Microwave-Crosslinked Polymer Binder MA-AANa/D Biodegradable in an Aqueous Environment–Selected Own Research

**DOI:** 10.3390/ma18235379

**Published:** 2025-11-28

**Authors:** Beata Grabowska, Artur Bobrowski, Mateusz Skrzyński, Grzegorz Grabowski, Wojciech Żyłka, Barbara Pilch-Pitera

**Affiliations:** 1 Faculty of Foundry Engineering, Department of Foundry Process Engineering, AGH University of Krakow, al. A. Mickiewicza 30, 30-059 Krakow, Poland; arturb@agh.edu.pl (A.B.); mateusz.skrzynski@agh.edu.pl (M.S.); 2 Faculty of Materials Science and Ceramics, Department of Ceramics and Refractories, AGH University of Krakow, al. A. Mickiewicza 30, 30-059 Krakow, Poland; grzegorz.grabowski@agh.edu.pl; 3 Faculty of Exact and Technical Sciences, Institute of Materials Engineering, University of Rzeszow, ul. Pigonia 1, 35-310 Rzeszow, Poland; wzylka@ur.edu.pl; 4 Faculty of Chemistry, Department of Polymers and Biopolymers, Rzeszow University of Technology, al. Powstańców Warszawy 12, 35-029 Rzeszow, Poland; barbpi@prz.edu.pl

**Keywords:** polymer, crosslinking, microwave, binder, biodegradation, foundry

## Abstract

The article presents a series of studies on a new polymer binder in the form of an aqueous composition of MA-AANa/D in the aspect of its reusability in the casting process and its safe storage in landfills. FT-IR analysis confirmed that microwave radiation induces crosslinking of both the MA-AANa/D binder and the moulding sand containing it. It was found that after simple treatment of the microwave-cured binder, its original binding properties can be restored, as the hydrogen-bond networks formed under microwave irradiation are reversible. The bending strength ($R_{g}^{u}$) tests of both fresh and regenerated moulding sands bonded with MA-AANa/D confirmed that the achieved curing degree meets the requirements for mould and core production. In addition, the biodegradability of the MA-AANa/D binder was evaluated using the Zahn-Wellens test. The progressive biodegradation was monitored through chemical oxygen demand (UV-Vis) measurements and the corresponding biodegradation degree (*R_t_*). The results confirmed that MA-AANa/D is fully biodegradable in aqueous environments, as evidenced by an *R_t_* value of 63.5% after 28 days of testing.

## 1. Introduction

The development of process and materials engineering is now driven by the use of environmentally friendly substrates, components, and materials that remain safe throughout their life cycle, including production, processing, use, and disposal. This trend results from tightening environmental regulations and the growing social awareness of ecological responsibility [1,2].

Throughout their life cycle, polymers and polymeric materials are continuously affected by physical (mechanical stresses, temperature, electromagnetic radiation), chemical (air components, oxygen), and biological (bacteria, enzymes) factors. Acting over time, these factors may induce both reversible and irreversible changes in their structure and physicochemical properties, ultimately leading to complete degradation. Understanding thenablenomena enables controlled use of these processes to achieve effects desirable for both industry and the environment [3,4,5].

In the foundry industry, environmental issues are primarily associated with harmful decomposition products generated during high-temperature casting processes. These originate from the organic binders (most commonly organic resins) and auxiliary additives (catalysts, hardeners, lustrous carbon additives) used in moulding and core sands [6,7,8,9,10]. Another important concern involves the storage of used moulding and core sands after casting, which must be treated as waste. Therefore, the selection of an appropriate binder for bonding the mineral matrix and the method of curing moulds and cores are key factors for sustainable foundry operations.

A properly selected binder and its curing mechanism should ensure the required bonding strength of the moulding or core sand, enable the production of high-quality castings, and at the same time exhibit minimal environmental impact throughout the entire technological process—from sand preparation, mould and core fabrication, and metal pouring to the handling of spent sands [6,11,12,13,14,15,16,17].

Literature reports describe the use of polymer-based and environmentally friendly biopolymer binders in foundry technologies [6,18,19,20,21,22]. The authors have been conducting extensive research in this area for several years [23,24,25,26,27]. However, the recycling of used moulding and core sands from a comprehensive perspective is rarely discussed in the literature. Both inorganic and organic binders that can regain their binding properties after suitable treatment are highly valued, and this aspect is also considered in the authors’ research [6,28,29,30].

In this regard, new polymeric binders of the BioCo group have been developed, containing polyacrylates and polysaccharides, which can be crosslinked thermally, by microwaves or UV radiation, or chemically using calcium hydroxide or glutaraldehyde [31,32]. Tests have confirmed that these binders do not negatively affect the natural environment [23].

Nevertheless, knowledge regarding the management of foundry waste remains incomplete. The biodegradation of binder residues in spent sands is still an open issue. This study aims to broaden understanding in this area by investigating a new polymer binder, MA-AANa/D (BioCo group), intended for mould and core production, with a focus on its recyclability and environmentally safe waste sand management.

## 2. Materials and Methods

### 2.1. Basic Materials

In this study, a polymer binder composed of a 35% aqueous solution of MA-AANa (maleic acid-acrylic acid copolymer sodium salt, BASF; M_n_ = 70,000 g/mol, pH 8, viscosity 2000 mPa·s) and a 40% aqueous solution of potato dextrin (Fluka). The polymer solutions were mixed in a 1:1 weight ratio to obtain a homogeneous and transparent composition. The formulation of this composition was established in previous systematic studies on BioCo binders [23].

The resulting aqueous polymer composition MA-AANa/D served as a binder for quartz matrix grains (silica sand: grain size fractions 0.20/0.16/0.32 mm, Sibelco, Bukowno, Poland). 

### 2.2. Crosslinking of the Binder

The crosslinking process of the MA-AANa/D binder (10 g) was carried out in a microwave field at a power of 800 W and a frequency of 2.45 GHz for 60, 90, and 120 s using an INOTEC MD 10940 microwave unit.(Medion AG, Essen, Germany) The selected crosslinking parameters were determined based on previous systematic studies on optimising the crosslinking of polyacrylates and their BioCo-based compositions [23]. Samples of the aqueous composition, both before and after crosslinking, were subjected to FT-IR structural analysis. To verify the reproducibility of network formation, another 10 g portion of MA-AANa/D was crosslinked again under identical conditions (800 W, 2.45 GHz, 60 s). After crosslinking, the sample was crushed, a stoichiometric amount of the released water was reintroduced, and the mixture was thoroughly mixed. The material was then crosslinked once more under the same microwave conditions (800 W, 2.45 GHz, 60 s). Each MA-AANa/D specimen, before and after crosslinking, was subsequently analysed by FT-IR spectroscopy.

### 2.3. FT-IR Structural Studies

Structural analyses were performed using a Digilab Excalibur FTS 3000 FT-IR spectrometer (Bio-Rad, Hercules, CA, USA), equipped with a standard DTGS detector, at a spectral resolution of 4 cm^−1^. Spectra were collected at room temperature in transmission mode using KBr pellets (potassium bromide for IR spectroscopy, Uvasol; Merck, Darmstadt, Germany).

### 2.4. Preparation of Moulding Sand

The moulding sand was prepared by mixing the components in a ribbon mixer (LM-R1)(Wadap, Marcyporęba, Poland): 100 parts by weight of silica sand and 3 parts by weight of the binder material. Mixing of the sand matrix with the free-flowing binder was continued for 3 minutes. The moulding sand was prepared at an ambient humidity of 25.1% and a temperature of 19 °C. From the prepared moulding sand, three series of standard longitudinal core samples with dimensions of 22.2 × 22.2 × 180 mm were produced according to the parameters required for the planned bending strength tests ($R_{g}^{u}$), in compliance with the PN-83/H-11073 standard [33]. Each series was subsequently cured in a microwave field.

### 2.5. Curing of Moulding Sand Samples

Three series of longitudinal core samples were subjected to microwave curing at a power of 800 W and a frequency of 2.45 GHz using an INOTEC MD 10940 microwave unit. The curing durations were 60 s (series 1), 90 s (series 2), and 120 s (series 3). Each series contained at least five samples. The longitudinal core samples were then subjected to bending strength tests in the cured state ($R_{g}^{u}$).

### 2.6. Bending Strength Testing of Cured Samples

The bending strength ($R_{g}^{u}$) of the three series of cured moulding sand samples was determined using a universal testing machine LRu-2e (Multiserw-Morek, Marcyporęba, Poland) within a measuring range of 0–870 N/cm^2^, in accordance with the PN-83/H-11073 standard.

Measurements of $R_{g}^{u}$ for standardised longitudinal samples made from both fresh and renewed sands were performed after 1 h and 24 h of storage in desiccators. The final bending strength value, considered as the actual result, was calculated as the arithmetic mean of at least five measurements from each series.

### 2.7. Process of Renewing the Moulding Sand 

The term renewed moulding sand is used in this study to denote cured moulding sand whose original binding properties were restored. After curing, the sand was mechanically disintegrated to obtain a free-flowing form. Its composition was then supplemented with an appropriate amount of water to dissolve the cured MA-AANa/D binder and restore its initial binding properties under microwave treatment.

The obtained mixture was homogenised for 3 minutes in a ribbon mixer (LM-R1). Standardised longitudinal samples were prepared from the renewed moulding sand, cured in a microwave field, and subsequently subjected to bending strength tests according to the methodology described in Section 2.5 and Section 2.6.

### 2.8. Investigations of Biodegradation in Aqueous Solution

The susceptibility of the MA-AANa/D polymer binder to biodegradation was evaluated using the static Zahn-Wellens test [34]. The biodegradation process was carried out in 1 dm^3^ vessels equipped with stirring and aeration systems. Each test mixture contained activated sludge (microorganisms), nutrient components (a basic bacterial medium), and aqueous solutions of the tested samples as the sole carbon source. Five samples were subjected to biodegradation: 1. Ethylene glycol (EG)—reference substance (expected biodegradation > 90%), POCh; 2. Polymer binder (MA-AANa/D); 3. Maleic acid-acrylic acid copolymer sodium salt (MA-AANa); 4. Potato dextrin (D); 5. Control sample (O) containing only activated sludge and nutrients (blank test). 

The activated sludge used in the control sample was obtained from the Water Supply System Department (MPWiK, Kraków, Poland). According to the standard procedure [34], the concentrations of the tested samples were maintained in the range of 50–400 mg/dm^3^. Since this method is intended for the assessment of organic substances, only the polymer binder was subjected to biodegradation testing, without the quartz matrix.

The incubation lasted 28 days and was carried out in the dark at an ambient temperature of 20–25 °C, with an aeration level of approximately 8 mg O_2_/dm^3^. Both temperature and aeration were continuously monitored throughout the test period.

The biodegradation process was further monitored at specified time intervals over the 28-day period by spectrophotometric determination of the chemical oxygen demand (COD) using the UV-Vis method, in accordance with the relevant standard procedure [35]. Changes in the degree of biodegradation (*R_t_*) of the samples were evaluated based on COD measurements following the Zahn-Wellens method. The *R_t_* values were calculated as the ratio of the COD value at a given time interval to the initial COD value, reflecting the progressive biodecomposition of the tested materials.

### 2.9. Microscopic Examinations

Microscopic observations were performed using a Nikon Eclipse LV100 optical microscope (Nikon Europe B.V., Amstelveen, The Netherlands) equipped with NIS-Elements AR 2.10 software. Images were recorded at a magnification of 50×.

## 3. Results

### 3.1. Microwave Crosslinking Process of the Polymer Binder 

Microwave radiation is used for curing foundry binders, primarily in moulding sand technologies based on hydrated sodium silicate. Research in this area is typically directed toward examining the absorption of electromagnetic waves in the microwave range (2.45 GHz) by uncured moulding sands bonded with silicates or aluminosilicates, including studies of their properties in the cured state [28,29,36,37,38,39,40]. In contrast, there is limited knowledge regarding the behaviour of organic binders under such conditions. Most organic binders in the form of resins exhibit hydrophobic properties, and their crosslinking proceeds via chemical reactions, often involving initiators, crosslinking agents, and catalysts.

In turn, aqueous polymer binders of the BioCo type, owing to their molecular structure and hydrophilic nature, are susceptible to microwave-induced crosslinking. They do not require any additional crosslinking agents, which makes them not only a cost-effective alternative to conventional resins but also environmentally friendly. However, the mechanism of microwave-induced crosslinking of hydrophilic polymers is still under investigation and continuously being developed by the research team. Understanding the chemistry of the polymer crosslinking process is essential for its further application in foundry technology—both in terms of the binder’s ability to bond mineral grains and its environmental assessment, which plays an important role in the casting process.

**Figure 1:**
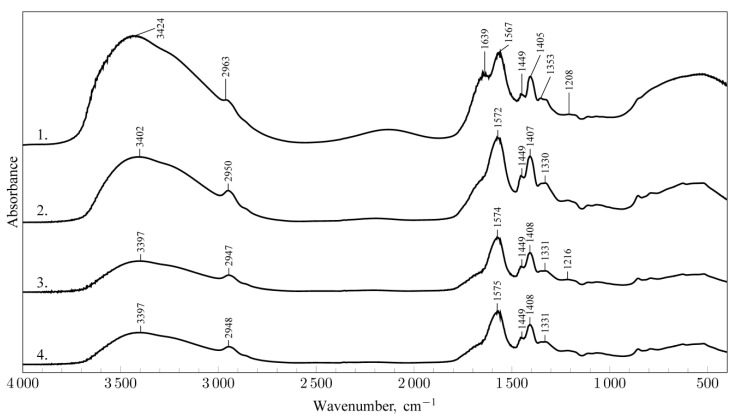
IR spectra of the MA-AANa/D composition: 1. initial state; 2. after microwave crosslinking (60 s); 3. after microwave crosslinking (90 s); 4. after microwave crosslinking (120 s).

In the first stage of the study, a structural analysis of the MA-AANa/D binder before and after microwave crosslinking was carried out using FT-IR spectroscopy (Figure 1). The crosslinking process under microwave radiation was performed at three exposure times: 60 s, 90 s, and 120 s.

The IR spectrum of the initial MA-AANa/D composition confirmed the presence of functional groups characteristic of its polymeric components—the synthetic polymer (maleic acid-acrylic acid copolymer sodium salt) and the biopolymer (dextrin). For the microwave-cured samples, noticeable changes were observed in the IR spectral profiles. The characteristic absorption bands identified in the IR spectra of the investigated system are summarised in Table 1.

It was found that exposure of the MA-AANa/D composition to microwave radiation for 60, 90, and 120 s induced structural transformations, manifested by changes in both the intensity and position of characteristic absorption bands. The broad band in the 3700–3000 cm^−1^ region changed its shape, and its maximum shifted toward higher wavenumbers. Additionally, the intensity of this band decreased as a result of microwave treatment, which is attributed to the evaporation of loosely bound (solvent) water. The persistence of the broad band, however, indicates a significant contribution of hydrogen bonding during the crosslinking process. Microwave electromagnetic radiation provides sufficient energy to promote rotational motion within polymer chains. As the absorbed energy propagates through the system, it activates polar functional groups (dipoles) capable of forming new chemical interactions. These interactions lead to the association of multiple polymer chains, resulting in the formation of numerous hydrogen bonds between hydroxyl (^...^O-H) and carbonyl (^...^O=C<) groups.

The spatially extended polymer networks formed through intra- and intermolecular hydrogen bonding significantly influence the binding strength of the crosslinked MA-AANa/D composition and, consequently, of the cured moulding sand in contact with quartz grains. Moreover, these bonds are physically reversible—upon reintroduction into water, they dissociate. This reversibility was also confirmed in the authors’ previous studies on the BioCo series of aqueous binders [23,30,31] and in the subsequent sections of this work.

A shift in the absorption band at 2963 cm^−1^ toward lower wavenumbers observed after microwave crosslinking may indicate changes in the position of C–H bonds located near polar groups involved in hydrogen bond formation. In contrast, the slight shifts observed around 1448 cm^−1^ and 1405 cm^−1^, originating from δ (CH_2_)_n_ deformation vibrations, fall within the measurement error range (±4 cm^−1^).

**Figure 2:**
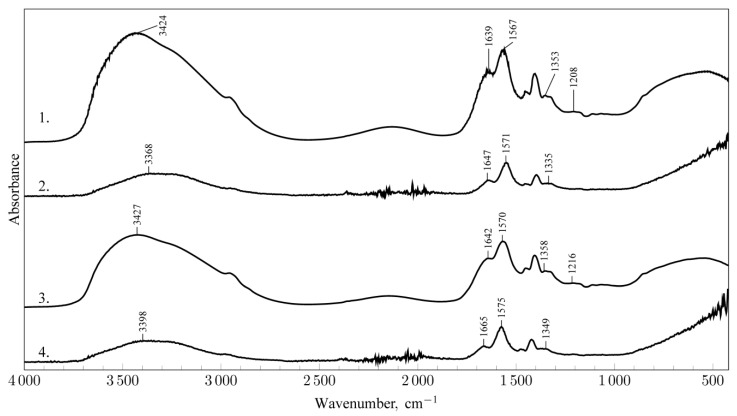
IR-Spectra MA-AANa/D: 1. initial state, 2. after cross-linking with microwaves (60 s), 3. after the renewal process, 4. after re-cross-linking with microwaves (60 s).

To demonstrate that the formed networks exhibit a reversible character and to assess the renewability of the binding properties of the crosslinked MA-AANa/D binder under microwave treatment, the material was successively subjected to dissolution in water and subsequent microwave crosslinking. For each binder state—before and after crosslinking—corresponding IR spectra were recorded (Figure 2).

In this context, the term renewability refers to the ability of the binder to restore its original binding properties after dissolution in water and to maintain its capacity for network formation under microwave radiation. Figure 2 presents the IR spectra of MA-AANa/D binder samples subjected to successive microwave crosslinking (60 s) and dissolution cycles.

After microwave crosslinking for 60 s, the broad absorption band in the 3700–3000 cm^−1^ region does not disappear (Figure 2, spectrum 2; cf. Figure 1, spectrum 2). However, its intensity decreases and the maximum shifts toward lower wavenumbers. These changes, as mentioned earlier, are related to the evaporation of loosely bound solvent water. The persistence of this band confirms the formation of hydrogen bonds within the crosslinked MA-AANa/D binder.

The band at approximately 1639 cm^−1^ (C–OH stretching vibrations) markedly decreases in intensity relative to the 1567 cm^−1^ band, consistent with the spectral patterns shown in Figure 1 (compare spectra 1 and 2). These bands are also shifted. Additionally, a reduction in the intensity of the band around 1333 cm^−1^ and its shift toward lower wavenumbers are observed after crosslinking. The band recorded near 1208 cm^−1^ also exhibits lower intensity. These spectral variations between spectra 1 and 2 indicate the involvement of polar functional groups in the crosslinking process.

The bands corresponding to C–H vibrations (–CH_2_ and –C–H groups) remain unchanged in position and intensity (bands: 1449 cm^−1^, 1405 cm^−1^). These groups are not relevant to the cross-linking process (compare Figure 1: the analysis of spectra 1–4). After redissolving the crosslinked MA-AANa/D binder in a stoichiometric amount of water, another IR spectrum was recorded (Figure 2, spectrum 3). Comparison of spectra 1 and 3 indicates that the MA-AANa/D binder retains its original structure. Re-exposure of the binder samples to microwave radiation results in spectral changes similar to those in spectrum 2 (Figure 2, compare spectra 2 and 4), confirming the re-crosslinking of the binder.

### 3.2. Strength and Renewability of Moulding Sands Bonded with the Polymer Binder

Previous studies on the strength of moulding sands bonded with BioCo binders based on polyacrylates and polysaccharides [23,30,31] demonstrated that the parameters of thermal curing (microwave exposure time and temperature) influenced the bending strength values. For BioCo binders, the use of microwave curing was found to provide faster and more efficient binder crosslinking, thereby making the method more cost-effective from the perspective of both process economics and practical foundry applications (e.g., in situ core curing).

**Figure 3:**
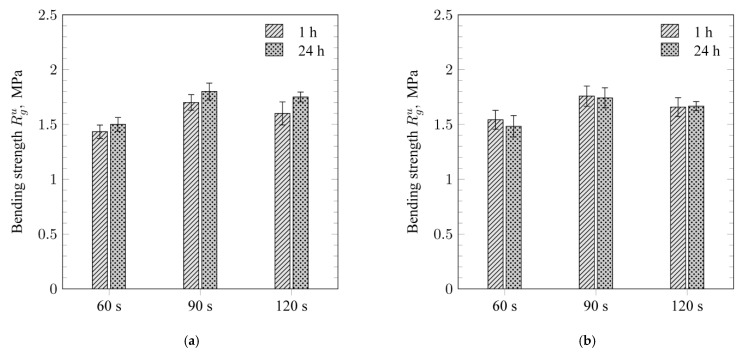
Bending strength R_g_^u^ of (**a**) fresh cured moulding sand and (**b**) renewed moulding sand after curing.

The bending strength ($R_{g}^{u}$) of samples prepared from fresh moulding sand and cured by microwaves for 60 s reached approximately 1.5 MPa (Figure 3a). The highest bending strength was obtained for samples cured for 90 s, with values around 1.7 MPa. Extending the microwave exposure time to 120 s did not significantly affect the strength values; in all cases, a slight decrease in $R_{g}^{u}\;$ was observed. 

Based on the obtained $R_{g}^{u}\;$ values, it can be concluded that the binder consisting of the sodium salt of a copolymer of maleic acid and acrylic acid and dextrin can be effectively cross-linked in the moulding sand under the influence of microwave radiation.

The bending strength $R_{g}^{u}$ of the renewed moulding sand also remained at approximately 1.5 MPa after microwave curing for 60 s (Figure 3b). The difference between the $R_{g}^{u}$ values obtained for the freshly cured and renewed moulding sands was within the measurement error range. Thus, it can be concluded that the MA-AANa/D binder retained its binding capacity after the regeneration process, and the optimal microwave curing time for the moulding sand was 90 s. These findings are consistent with the results of the structural analyses of the MA-AANa/D binder before and after crosslinking. Both in the binder and in the binder applied to the mineral matrix (i.e., in the cured moulding sand), microwave radiation induced the formation of hydrogen-bonded networks whose strength was sufficient for producing moulds and cores intended for casting applications [6].

An important observation is the ability of the MA-AANa/D binder to restore its binding properties within the moulding sand. Spent moulding sands from the casting process, after removal of the surface layer thermally affected by contact with molten metal, can be reintroduced into production with minimal treatment. The thermally unaltered binder present in the spent sand can be redissolved in water and re-crosslinked. This feature also supports safer storage and management of foundry waste. Therefore, in the next stage of this study, the biodegradability of the MA-AANa/D binder in an aqueous environment was evaluated.

### 3.3. Biodegradation of the Polymer Binder

The biodegradation process was carried out for five samples: 1. Ethylene glycol (EG)—reference compound (expected biodegradation > 90%), POCh; 2. Polymer binder (MA-AANa/D); 3. Maleic acid-acrylic acid copolymer sodium salt (MA-AANa); 4. Potato dextrin (D); 5. Control sample (O), as described in Section 2.8.

**Figure 4:**
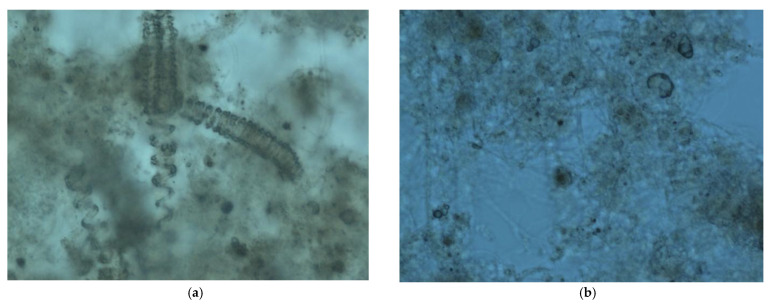
Examples of microscopic photographs of microorganisms for the reference sample: (**a**) 2 days after sampling, (**b**) 20 days after sampling.

In addition to the reference (EG) and control (O) samples, aqueous solutions of the individual polymers forming the MA-AANa/D binder were also examined to provide additional insight into the biodegradation behaviour of the starting components. Throughout the 28-day biodegradation process, the environmental parameters necessary for proper microbial activity were continuously controlled. The temperature was maintained within the recommended range of 20–25 °C, and the aeration level of the tested samples was kept at approximately 8 mg O_2_/dm^3^. To confirm the sustained presence of viable microorganisms in the mixtures, periodic microscopic observations of the samples were performed (Figure 4). Microorganisms were observed in accordance with the accepted methodology for illustrative purposes. In addition, the biodegraded samples were characterised (colour, odour, structure and concentration of flocs, biodiversity of microorganisms, clarity, sedimentation). The presence of live microorganisms in the sample taken at the beginning of the test (Figure 4a) and at the end of the test, as confirmed by microscopic examination (Figure 4b), provides additional evidence that the conditions for the biodegradation process were appropriate (in accordance with the standard) and did not adversely affect their natural development and metabolism in the presence of the polymers D and MA-AANa and the MA-AANa/D composition present in the tested samples.

During the biodegradation test, the chemical oxygen demand (COD) was determined spectrophotometrically (UV-Vis) for all analysed, mixtures. In accordance with the test requirements, COD values were measured at selected time intervals. The decrease in COD values over time, compared with the value determined three hours after the start of the test, was used to calculate the percentage degree of biodegradation of the samples (Table 2).

Based on the obtained COD values, the percentage of degree of biodegradation (*R_t_*) was determined for the tested samples: EG, D, MA-AANa, and MA-AANa/D. The COD values were calculated according to Equation (1):

(1) $$R_{t}(\%)\;=\;\left[1-\frac{\left(C_{T}-C_{B}\right)}{\left(C_{A}-C_{BA}\right)}\right]\cdot 100\%$$

where:

*R_t_*—Biodegradation % in time (within 28 days),
*C_A_*—COD values in the tested mixture, determined 3 hours after the start of the experiment (mg/dm^3^),
*C_T_*—COD values in a mixture during sampling (mg/dm^3^),
*C_B_*—COD values in a blank test during sampling (mg/dm^3^),
*C_BA_*—COD values in a blank test, determined 3 hours after the beginning of the experiment (mg/dm^3^).

**Figure 5:**
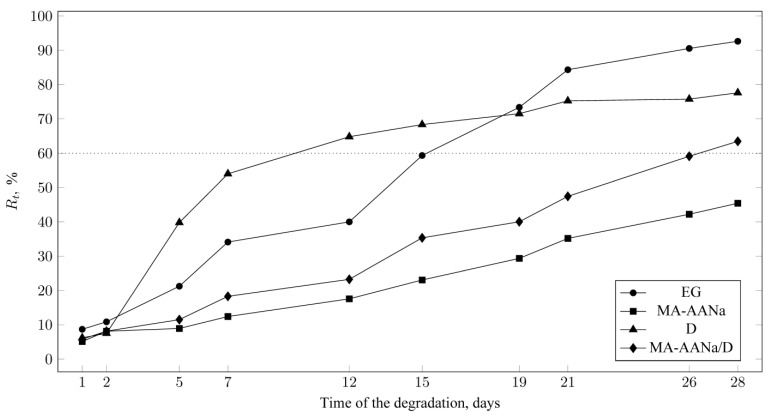
Biodegradation curves for samples EG, MA-AANa, D and MA-AANa/D.

The percentage degree of biodegradation was determined from the relative decrease in the COD value over time. The calculated *R_t_* values are presented in the graph as a function of biodegradation time, forming the biodegradation curve (Figure 5).

A polymer is considered biodegradable when its degree of biodegradation after a 28-day test cycle reaches at least 60% [28]. This condition was met by the following samples: the reference sample, ethylene glycol (*R_t_* = 93%), dextrin (D) (*R_t_* = 77%), and the MA-AANa/D binder (*R_t_* = 63%). Although the presence of the synthetic polymer component, maleic acid-acrylic acid copolymer sodium salt (MA-AANa), hindered the biodegradation process, the binder reached the required *R_t_* value after 28 days, allowing it to be classified as fully biodegradable. 

The biodegradation test performed on the MA-AANa polymer alone confirmed its limited susceptibility to biological degradation, with an *R_t_* value of 45%, excluding it from the group of fully biodegradable polymers in aqueous environments. The presence of sodium ions hinders the biodegradation process; however, it simultaneously improves its water solubility and results in a more favourable pH for interaction with mineral grains during bonding.

Consequently, the final aqueous polymer composition exhibits superior properties compared with its individual components, MA-AANa and D. The proportion of dextrin in the composition allows the resulting MA-AANa/D binder to be classified as biodegradable.

Based on the biodegradation results for the MA-AANa/D binder, together with the evidence of its renewability in spent sands, it can be concluded that the binder present in used sands will gradually biodegrade during storage under ambient conditions at landfill sites.

## 4. Conclusions 

The binder composed of maleic acid-acrylic acid copolymer sodium salt and dextrin can be effectively crosslinked in an aqueous composition under microwave radiation. The microwave crosslinking process is physically reversible: dissolution of the crosslinked binder in water restores its initial structure by releasing loosely bound solvent water. No chemical reactions occur within the functional groups during crosslinking. The resulting hydrogen-bonded network originates from dipole–dipole interactions between polar polymer groups (hydroxyl and carbonyl). FT-IR analyses confirmed the renewability of the MA-AANa/D binder.

The obtained $R_{g}^{u}$ bending strength values indicate that the aqueous binder, composed of the sodium salt of a maleic acid-acrylic acid copolymer and dextrin, can be effectively crosslinked in moulding sand under microwave radiation.

The renewed moulding sand exhibited bending strength comparable to that of the freshly cured sand. From a technological perspective, the achieved bending strength values are sufficient for proper mould and core fabrication.

The biodegradation of foundry polymer binders under microbial influence is a complex process, dependent both on the binder’s chemical structure—particularly the presence of polar hydroxyl and carboxylate groups—and on environmental factors such as temperature, pH stability, nutrient availability, oxygen concentration, and humidity. Based on chemical oxygen demand results, the MA-AANa/D binder underwent gradual biodecomposition, reaching a degree of biodegradation of *R_t_* = 63% after 28 days. According to reference criteria, this value qualifies the material as fully biodegradable. The findings provide valuable insight into the potential behaviour and degradation pathway of polymeric binders under landfill storage conditions.

## Figures and Tables

**Table 1:** Characteristic bands in the IR spectra.

Spectra 1	Spectra 2	Spectra 3	Spectra 4	Assignment	Remarks
Wavenumber, cm^−1^					
3424	3402	3397	3397	ν-OH O-H^...^O-H O-H^...^O=C	Band of free OH group Hydrogen bond (between hydroxyl groups) Hydrogen bond (between hydroxyl and carbonyl groups)
2963 -	- 2950	- 2947	- 2948	ν-C-H	Stretching vibrations asymmetric and symmetric
1639	-	-	-	ν_as_-COO^−^	Stretching asymmetric vibration carboxylate group
1567 -	- 1572	- 1574	- 1575	δ-C-O-H	Deforming vibrations hydroxyl group
1448 1405	1449 1407	1449 1408	1449 1408	δ (CH_2_)_n_	Shearing symmetric vibrations
1353 -	- 1330	- 1331	- 1331	ν_s_-COO^−^	Stretching symmetric vibration carboxylate group
1208 -	- 1216	- 1216	- 1216	C-O-H	Deforming vibrations

**Table 2:** Chemical oxygen demand (COD) values for the tested samples.

Sample Measurement (Day)	EG (Standard)	D	MA-AANa	MA-AANa/D	O (Blank Test)
	COD, mg/dm^3^ O_2_				
3 h	1245.5	685.1	768.5	754.6	9.752
1 (1)	1138.1	620.5	729.8	711.4	9.820
2 (2)	1107.5	570.3	703.1	689.9	6.032
3 (5)	979.6	403.7	697.6	665.3	6.545
4 (7)	820.5	310.1	670.9	614.7	6.346
5 (12)	748.1	239.0	632.1	578.2	6.670
6 (15)	508.9	215.4	584.1	487.1	6.532
7 (19)	335.1	194.2	542.4	453.0	6.313
8 (21)	200.6	170.2	498.9	398.5	7.017
9 (26)	125.0	168.1	446.7	312.7	8.315
10 (28)	100.2	156.9	423.1	281.1	9.130

## Data Availability

The original contributions presented in this study are included in the article. Further inquiries can be directed to the corresponding author.
